# Circular RNAs expression profiles in human gastric cancer

**DOI:** 10.1038/s41598-017-09076-6

**Published:** 2017-08-22

**Authors:** Yuan Dang, Xiaojuan Ouyang, Fan Zhang, Kai Wang, Youdong Lin, Baochang Sun, Yu Wang, Lie Wang, Qiaojia Huang

**Affiliations:** 1 Department of Experimental Medicine, Fuzhou General Hospital, 156 North Xi-er Huan Road, Fuzhou City, Fujian Province 350025 China; 2 Department of General Surgery, Fuzhou General Hospital, 156 North Xi-er Huan Road, Fuzhou City, Fujian Province 350025 China

## Abstract

Circular RNAs (circRNAs) are implicated in a variety of cancers. However, the roles of circRNAs in gastric cancer (GC) remain largely unknown. In the current study, circRNAs expression profiles were screened in GC, using 5 pairs of GC and matched non-GC tissues with circRNA chip. Preliminary results were verified with quantitative PCR (qRT-PCR). Briefly, total of 713 circRNAs were differentially expressed in GC tissues vs. non-GC tissues (fold change ≥ 2.0, p < 0.05): 191 were upregulated, whereas 522 were downregulated in GC tissues. qRT-PCR analysis of randomly selected 7 circRNAs from the 713 circRNAs in 50 paired of GC vs. non-GC control tissues confirmed the microarray data. Gene ontology (GO) and KEGG pathway analyses showed that many circRNAs are implicated in carcinogenesis. Among differentially expressed circRNAs, hsa_circ_0076304, hsa_circ_0035431, and hsa_circ_0076305 had the highest magnitude of change. These results provided a preliminary landscape of circRNAs expression profile in GC.

## Introduction

Gastric cancer (GC) is one of the most common cancers worldwide^1^. Diagnosis and treatment have improved over the last decades, but the 5-year survival rate remains low in patients with advanced GC^2^. A lack of reliable and efficient early diagnostic biomarkers, as well as, poorly understood molecular mechanisms of this disease is a major factor. To improve patient outcome, identifying effective biomarkers with early diagnostic value is essential. Novel biomarkers may also reflect the characteristics of cancer and clarify the molecular mechanisms of GC.

Over the past decade, the roles of non-coding RNA in cancer have been under intense investigation, encompassing miRNAs to long non-coding RNAs (lncRNA) and recently identified circular RNAs (circRNAs)^3, 4^. Accumulating evidence has demonstrated that both miRNAs and lncRNAs are closely associated with human cancers; many play crucial roles in cancer progression. Recent studies have implicated circRNAs in cancer development^5^. However, there have been relatively few reports describing circRNAs in GC.

CircRNAs are novel circular non-coding RNAs that are covalently closed^6^. CircRNAs could mediate the activity of microRNAs through binding and functioning as their sponges. Increasing evidence has suggested that circRNAs are often abnormally expressed in human cancers, and contribute to oncogenesis through miRNAs^7^. CircRNAs regulate cancer-related pathways and linear RNA transcription as well as protein expression^8, 9^. However, the expression levels and potential roles of circRNAs in GC are still poorly understood. In the present study, we investigated the alteration of circRNA expression profiles in GC tissues.

## Materials and Methods

### Tissue samples

A total of 55 patients (44 men and 11 women; mean age 59.8 years with a range of 23–81) with GC who underwent radical resection of the primary lesions between June 2014 and May 2015 at the Fuzhou General Hospital were included in this study. All tissues were histologically identified, diagnosed as gastric adenocarcinoma, and graded according to the guidelines of modified American Joint Committee on Cancer (AJCC). The initial screening step (Table 1) was conducted with microarray chip assay in 5 pairs of GC vs. non-GC tissue sample; the remaining 50 pairs were used for verification with quantitative reverse transcription PCR (qRT-PCR). Prior to analysis, all tissue samples were processed using a previously published method^10^ and stored at −80 °C.

**Table 1:** The information of patients with gastric cancer subjected to circRNA expression profile chip assay.

NO	Gender (male or female)	Age (years)	Histological type	Histologic differentiation	TNM stage
286	M	74	Ulcerative	Moderately	T2N0Mx
287	M	74	Ulcerative	Poorly	T4aN1Mx
292	M	78	Ulcerative	Moderately	T4aN2Mx
313	M	58	Ulcerative	Moderately-poorly	T2N0Mx
326	M	61	Ulcerative	Moderately	T4aN0Mx

### RNA preparation for chip assay

TRIzol reagent (Invitrogen, Carlsbad, CA, USA) and mirVana miRNA Isolation Kit (Ambion, Austin, TX, USA) were used to isolate and purify total RNA. Sample quality (purity) was verified using standard spectrophotometer (ND-1000). The RNA integrity was assessed by electrophoresis with denaturing agarose gel.

### Labeling and hybridization

After removing linear RNAs with ribonuclease R, RNA (5 μg from each sample) was reverse transcribed into cDNA using random primers containing T7 promoter by First Strand Enzyme Mix Kit. The DNA-RNA mixture was transformed to the second strand DNA by Second Strand Enzyme Mix. This DNA was used as a template to synthesize cRNA by the T7 enzyme mix. Subsequently, the cRNA was used as a template to obtain cDNA by reverse transcription through CbcScript II enzyme combined with random primers. This cDNA, in turn, was used as a template to synthesize the complementary strand DNA labeled fluorescently by Klenow Fragment enzyme combined with random primers and dNTP with fluorescent tags such as Cy3-dCTP and Cy5-dCTP. The samples were then hybridized with a  CapitalBio Technology Human CircRNA Array v1 (Agilent, USA).

Signals were scanned by Agilent G2565CA Microarray Scanner. Images were introduced into Agilent Feature Extraction to obtain raw data (v10.7). Differential expression was analyzed with Agilent GeneSpring software, and the processing included raw data quantile normalization and data analysis. Post quantile normalization by log2-ratio, low-intensity filtering was conducted, and circRNAs with at least 60 percent samples flagged as “Detected” were selected for further analysis: differentially expressed circRNAs were analyzed with Independent samples *t*-test. CircRNAs with ≥2.0 fold-changes (FC) and p < 0.05 were selected as circRNAs with significant differential expression^11^.

### Bioinformatics analysis

circRNA targets identified with profiling data were subjected to gene ontology (GO) and KEGG pathway analyses based on their correlated mRNAs using Gene Ontology (http://www.geneongoloty.org/) and KOBAS software (KEGG Orthology-Based Annotation System). The differentially expressed circRNAs-targeted miRNAs were sought and predicted by miRanda software coupled with statistical analysis. In order to understand the association between circRNAs and their related miRNAs, 3 most significantly altered circRNAs were used to draw the circRNA-miRNA network using miRanda combined with patterning software. The circRNAs expression profile microarray chip assay and data and bioinformatics analysis were carried out by Capitalbio Corporation (Beijing, China).

### qRT-PCR assay

Total RNA was extracted by TRIzol reagent as described previously^10^. The expression levels of 7 randomly selected differentially expressing circRNAs (Fold changes ≥ 2, p < 0.05) were measured by qRT-PCR; among them, 2 were upregulated and 5 were downregulated in the GC tissues: (upregulated: hsa_circ_0081146, hsa_circ_0084720), (downregulated: hsa_circ_0060108, hsa_circ_0057104, hsa_circ_0054971, hsa_circ_0063561, and hsa_circ_0058766). GAPDH expression was used as an internal reference. The primers used for these amplifications are listed in Table S1. PCRs were a relative estimation in triplicate as per the following temperature profile:denaturation 95 °C for 10 min followed by amplification by 40 cycles of 95 °C for 10 s and 60 °C for 1 min^10^.

### Statistical analysis

For comparisons involving multiple groups, data were analyzed by analysis of variance (ANOVA); For analysis involving only two groups, data were analyzed with Student’s *t*-test. Results are expressed as the mean ± SEM. p < 0.05 was regarded as statistically significant. Data analysis was performed by Statistical Program for Social Sciences (SPSS) 22.0 software (SPSS, Chicago, IL, USA).

### Compliance with ethical standards

The tissue samples used in this study were obtained with patients informed consent. ﻿All the methods were performed in compliance with the permitted or institutional protocols.This study was approved by the Fuzhou General Hospital Ethics Committee (No. 2014CXTD04). This article does not contain any studies with animals performed by any of the authors.

## Results

### CircRNAs expression profiles in GC

**Figure 1:**
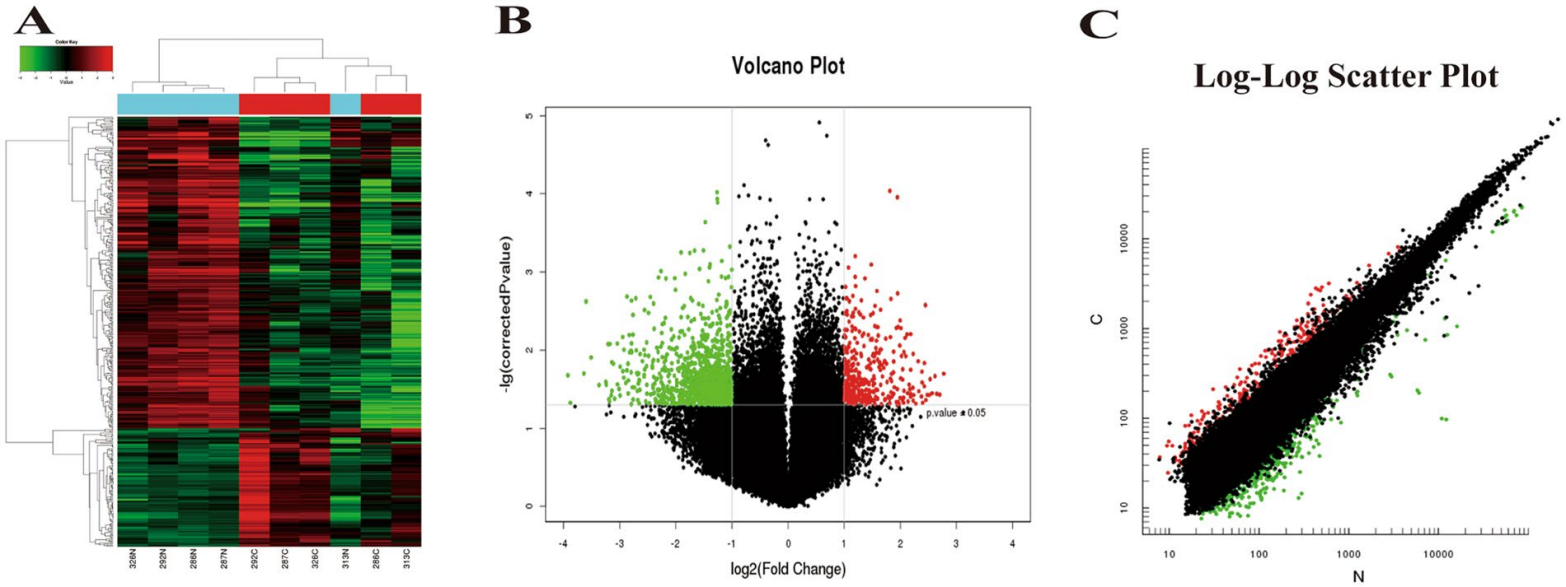
Hierarchical clustering, volcano plots, and scatter plots exhibited the differentially expressed circRNAs in gastric cancer tissues compared to paired non-gastric cancer tissues. (**A**) Hierarchical clustering, numbers were the samples used for the microarray assay. C: cancer tissues, N: non-cancerous tissues. (**B**) Differentially expressed circRNAs were displayed by volcano plots. The green and red parts indicated >2 fold-decreased and -increased expression of the dysregulated circRNAs in GC tissues, respectively (p < 0.05). (**C**) Differentially expressed circRNAs were displayed by scatter plots. The green and red parts indicated >2 fold-decreased and -increased expression of the dysregulated circRNAs in GC tissues (p < 0.05).

The microarray screening detected a total of 62,998 circRNAs, in GC, non-GC or both tissues (such information could be accessed with GSE100170 at https://www.ncbi.nlm.nih.gov/geo/query/acc.cgi?acc = GSE100170). As illustrated in Fig. 1, 713 of these exhibited differential expressions between GC and non-GC tissues (FC ≥ 2.0, p < 0.05) (Table S2), among which 191 were upregulated and the remaining 522 were downregulated in cancer tissues. A total of 207 circRNAs were differentially expressed between GC and non-GC tissues by both long and short probes (the two kinds of probe were named CBC1 and CBC2, respectively), among which 57 were upregulated and 150 were downregulated. The magnitude of fold change was highest for hsa_circ_0044516 in upregulated circRNAs (fold changes = 6.28, p = 0.036), and hsa_circ_0076305 for downregulated circRNAs (fold changes = −125.95, p = 0.030). Hierarchical clustering (Fig. 1A), volcano plots (Fig. 1B), and scatter plots (Fig. 1C) revealed that the expression profiles of circRNAs between GC and non-GC tissues were diverse. The top up- and down-regulated circRNAs are displayed in Table 2.

**Table 2:** The top up- and down-regulated differentially expressed circRNAs in GC tissues compared to those in non-cancerous tissues by both probes.

Name	Probe CBC1			Probe CBC2			chr	gene symbol
	p	FC (abs)	Regulation	p	FC (abs)	Regulation		
hsa_circ_0076305	0.029925665	125.95259	down	0.03022709	109.61993	down	chr6	PGC
hsa_circ_0076304	0.014676455	31.56073	down	0.011826442	28.374382	down	chr6	PGC
hsa_circ_0035431	0.00300389	20.631256	down	0.003938972	21.39568	down	chr15	CGNL1
hsa_circ_0000390	0.01983777	12.444183	down	0.026148466	7.417858	down	chr12	FGD4
hsa_circ_0037362	0.027888432	10.314745	down	0.014241457	8.81237	down	chr16	C16orf73
hsa_circ_0076307	0.025423396	9.498694	down	0.020947674	15.158471	down	chr6	PGC
hsa_circ_0037361	0.027664987	9.436992	down	0.002388634	12.108136	down	chr16	C16orf73
hsa_circ_0007315	0.042465463	9.14959	down	0.03788745	8.887227	down	chr3	PVRL3
hsa_circ_0027969	0.014839446	8.641936	down	0.03794105	7.963011	down	chr12	SLC41A2
hsa_circ_0001679	0.031237341	8.059054	down	0.04918231	6.926098	down	chr7	GLCCI1
hsa_circ_0035435	0.014802514	7.2164187	down	0.045140408	5.798211	down	chr15	CGNL1
hsa_circ_0027971	0.03575782	7.201395	down	0.008316714	9.217224	down	chr12	SLC41A2
hsa_circ_0073770	0.008143293	6.911025	down	0.006314759	6.7912025	down	chr5	SLC12A2
hsa_circ_0074239	0.002184121	6.5593286	down	0.003311638	5.6043277	down	chr5	C5orf32
hsa_circ_0051995	0.020385692	6.4125986	down	0.027742783	5.7211037	down	chr19	VRK3
hsa_circ_0025842	0.024139885	6.305559	down	0.019139148	6.9465156	down	chr12	FGD4
hsa_circ_0077736	0.048207846	6.2890162	down	0.033191722	6.435054	down	chr6	CEP85L
hsa_circ_0006034	0.01381651	6.038208	down	0.003529549	6.7874093	down	chr5	SLC12A2
hsa_circ_0066971	0.011570533	5.8554688	down	0.036805384	5.452018	down	chr3	EAF2
hsa_circ_0035432	0.011778097	5.5990286	down	0.011175622	6.2109637	down	chr15	CGNL1
hsa_circ_0044516	0.036079686	6.276136	up	0.04336383	5.479236	up	chr17	COL1A1
hsa_circ_0044518	0.037375137	5.72242	up	0.04342119	3.3064938	up	chr17	COL1A1
hsa_circ_0077033	0.018847013	5.221709	up	0.023423905	5.7803392	up	chr6	COL12A1
hsa_circ_0006401	0.013833045	4.9873238	up	0.017758103	4.545301	up	chr2	COL6A3
hsa_circ_0081090	0.036175337	4.5390043	up	0.021447672	6.136692	up	chr7	COL1A2
hsa_circ_0058132	0.045273677	4.3983254	up	0.022904716	4.0176225	up	chr2	FN1
hsa_circ_0081146	0.010543266	4.187957	up	0.011812083	4.0625715	up	chr7	COL1A2
hsa_circ_0058100	0.047036752	4.166924	up	0.04413781	4.6881175	up	chr2	FN1
hsa_circ_0081091	0.008671624	3.9734251	up	0.014162468	3.2844515	up	chr7	COL1A2
hsa_circ_0091742	0.005013032	3.8982337	up	0.014553196	5.383641	up	chrX	BGN
hsa_circ_0058097	0.049916536	3.8704908	up	0.033016972	4.187093	up	chr2	FN1
hsa_circ_0081136	0.013954006	3.6851623	up	0.021678727	3.2075522	up	chr7	COL1A2
hsa_circ_0044519	0.03606566	3.6805367	up	0.03702461	6.5064387	up	chr17	COL1A1
hsa_circ_0016294	0.034532506	3.5717776	up	0.038935255	3.4514186	up	chr1	CD55
hsa_circ_0081143	0.002223194	3.5639381	up	0.03638	3.226277	up	chr7	COL1A2
hsa_circ_0044515	0.048010923	3.5043917	up	0.047074426	5.2711334	up	chr17	COL1A1
hsa_circ_0081066	0.040320095	3.4538522	up	0.006778345	3.5394926	up	chr7	COL1A2
hsa_circ_0016292	0.016833305	3.2611954	up	0.017872557	3.1238286	up	chr1	CD55
hsa_circ_0091743	0.015532353	3.1929755	up	0.006474511	3.045686	up	chrX	BGN
hsa_circ_0020788	0.005037079	3.030931	up	0.005184689	3.8050945	up	chr11	TCONS_00063837_H19

FC: Fold changes. abs: absolute value.

### The results of qRT-PCR verification of the differentially expressed circRNAs

**Figure 2:**
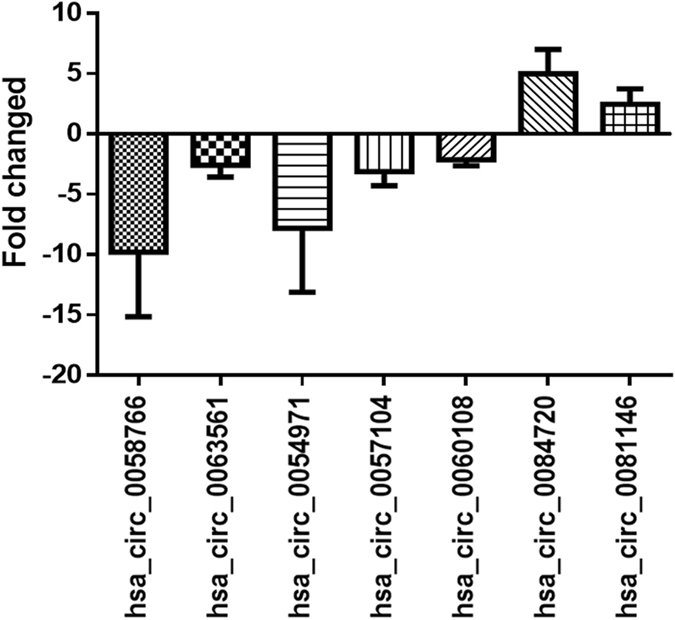
Verification of the differentially expressed circRNAs by qRT-PCR. The expression of 7 lncRNAs in 50 paired GC tissues was detected by qRT-PCR, which were shown by the expression fold changes. Comparison of the results obtained from qPCR and microarray assay revealed satisfactory consistency.

Seven differentially expressed circRNAs were randomly selected for qRT-PCR verification  by using 50 paired of samples. The results confirmed the upregulation of hsa_circ_0081146 and hsa_circ_0084720 in GC, and downregulation of hsa_circ_0060108, hsa_circ_0057104, hsa_circ_0054971, hsa_circ_0063561, and hsa_circ_0058766 in GC (Fig. 2).

### The results of bioinformatics analysis

**Figure 3:**
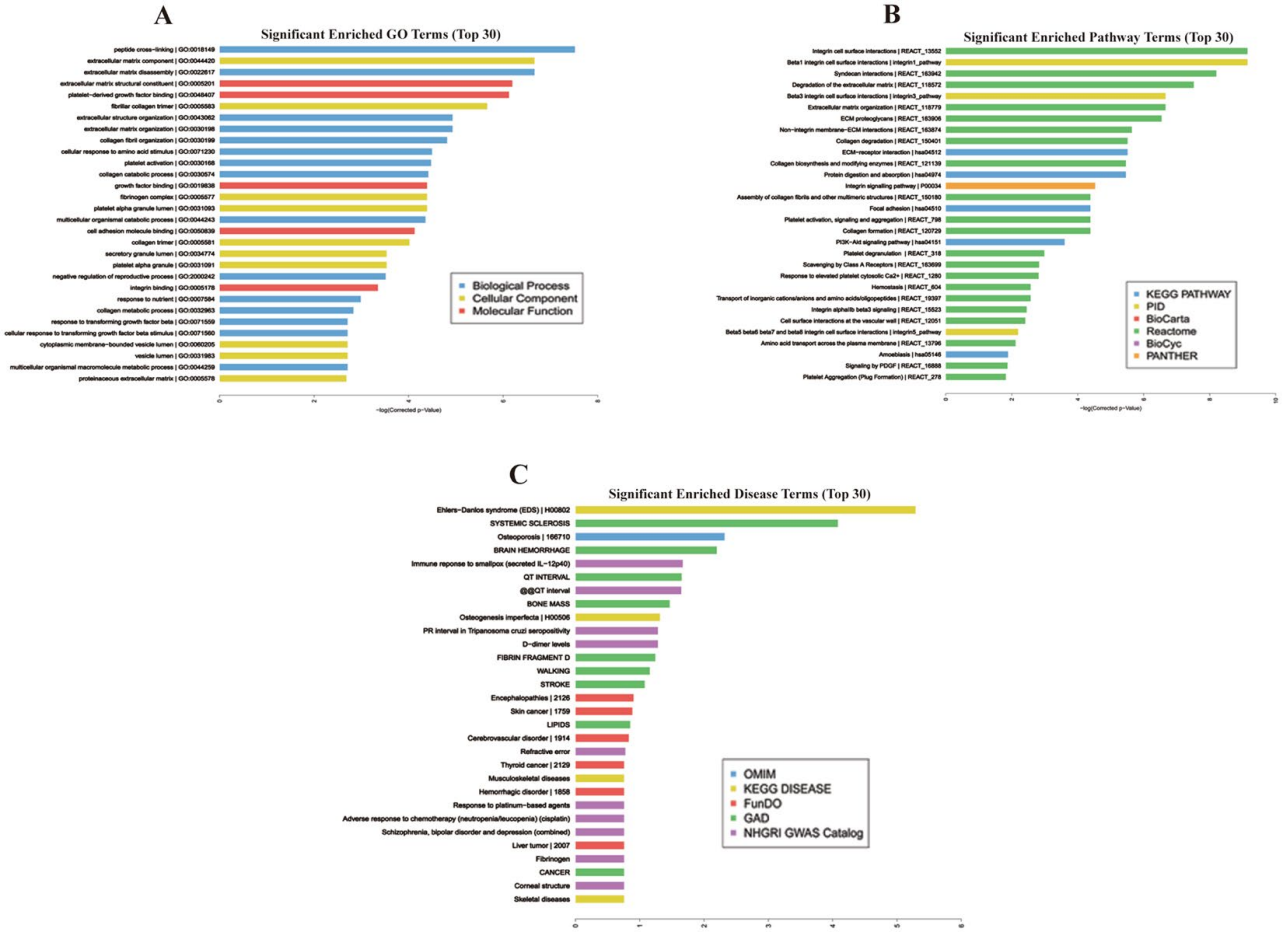
Results of Gene Ontology, KEGG pathway, and disease enrichment analysis. (**A**) Top 30 classes of GO enrichment terms. **(B)** Top 30 classes of KEGG pathway enrichment terms. **(C)** Top 30 disease enrichment terms.

Differentially expressed circRNAs could be mapped to all chromosomes, except for chromosome 21 and Y. A lot of miRNAs were predicted to be their targets (Table 3). 1026 miRNAs were predicted to be the targets of hsa_circ_0001210, which is an intragenic circRNA, located on chromosome 22 with a length of 25285 nt and downregulated in GC. 116 mRNAs were shown to be the potential corresponding linear transcripts of these dysregulated circRNAs (Table S3). GO, KEGG, and enrichment (Table S4) analyses suggest that these differentially expressed circRNAs are relevant to several vital physiological processes, cellular components, molecular functions, and critical signaling pathways such as growth factor binding, cell adhesion molecule binding, and response to transforming growth factor beta (TGF-β). Many of the known pathways associated with carcinogenesis, such as focal adhesion pathway, PI3K–Akt signaling pathway, and degradation of the extracellular matrix pathway were also implicated. Figure 3A–C illustrated the top 30 significantly enriched GO terms, pathway terms, and disease terms.

**Table 3:** The numbers of potential targeted miRNAs of the differentially expressed circRNAs.

ProbeName	p	FC (abs)	Regulation	chr	gene symbol	No. miRNA targets
hsa_circ_0006401	0.013833045	4.9873238	up	chr2	COL6A3	17
hsa_circ_0014202	0.008794556	2.5519805	up	chr1	S100A10	4
hsa_circ_0016292	0.016833305	3.2611954	up	chr1	CD55	0
hsa_circ_0016294	0.034532506	3.5717776	up	chr1	CD55	0
hsa_circ_0018424	0.004929639	2.2052047	up	chr10	BICC1	1
hsa_circ_0020788	0.005037079	3.030931	up	chr11	TCONS_00063837_H19	0
hsa_circ_0020790	0.002316416	2.4726825	up	chr11	TCONS_00063837_H19	0
hsa_circ_0034428	0.03272182	2.6071007	up	chr15	THBS1	87
hsa_circ_0034475	0.028014906	2.2003295	up	chr15	THBS1	23
hsa_circ_0034495	0.018290553	2.2112164	up	chr15	THBS1	3
hsa_circ_0034496	0.023052732	2.150319	up	chr15	THBS1	8
hsa_circ_0035137	0.026556138	2.3628845	up	chr15	FBN1	1
hsa_circ_0044513	0.044563152	2.5272765	up	chr17	COL1A1	46
hsa_circ_0044515	0.048010923	3.5043917	up	chr17	COL1A1	180
hsa_circ_0044516	0.036079686	6.276136	up	chr17	COL1A1	191
hsa_circ_0044517	0.03509533	2.7368069	up	chr17	COL1A1	230
hsa_circ_0044518	0.037375137	5.72242	up	chr17	COL1A1	245
hsa_circ_0044519	0.03606566	3.6805367	up	chr17	COL1A1	267
hsa_circ_0044521	0.03614439	2.4449718	up	chr17	COL1A1	176
hsa_circ_0046707	0.033796836	2.1029172	up	chr18	SMCHD1	5
hsa_circ_0057391	0.04772664	2.256704	up	chr2	COL3A1	193
hsa_circ_0057403	0.03509203	2.146545	up	chr2	COL3A1	46
hsa_circ_0058097	0.049916536	3.8704908	up	chr2	FN1	159
hsa_circ_0058100	0.047036752	4.166924	up	chr2	FN1	56
hsa_circ_0058132	0.045273677	4.3983254	up	chr2	FN1	9
hsa_circ_0077033	0.018847013	5.221709	up	chr6	COL12A1	13
hsa_circ_0077055	0.038407695	2.5744588	up	chr6	COL12A1	0
hsa_circ_0077056	0.04552718	2.9007647	up	chr6	COL12A1	1
hsa_circ_0077057	0.04089677	2.6701639	up	chr6	COL12A1	0
hsa_circ_0080229	0.03741656	2.027931	up	chr7	EGFR	0
hsa_circ_0081066	0.040320095	3.4538522	up	chr7	COL1A2	243
hsa_circ_0081084	0.032507733	2.7151222	up	chr7	COL1A2	136
hsa_circ_0081089	0.03845984	2.0764067	up	chr7	COL1A2	163
hsa_circ_0081090	0.036175337	4.5390043	up	chr7	COL1A2	227
hsa_circ_0081091	0.008671624	3.9734251	up	chr7	COL1A2	260
hsa_circ_0081092	0.044405155	2.7602344	up	chr7	COL1A2	4
hsa_circ_0081111	0.005212786	4.0334992	up	chr7	COL1A2	237
hsa_circ_0081125	0.013989674	2.8943481	up	chr7	COL1A2	182
hsa_circ_0081136	0.013954006	3.6851623	up	chr7	COL1A2	156
hsa_circ_0081137	0.039436605	2.9486153	up	chr7	COL1A2	112
hsa_circ_0081138	0.047790088	2.2264013	up	chr7	COL1A2	121
hsa_circ_0081143	0.002223194	3.5639381	up	chr7	COL1A2	89
hsa_circ_0081146	0.010543266	4.187957	up	chr7	COL1A2	121
hsa_circ_0081149	0.045875933	2.6191776	up	chr7	COL1A2	69
hsa_circ_0081152	0.011682078	2.843584	up	chr7	COL1A2	99
hsa_circ_0081155	0.00731891	2.8246753	up	chr7	COL1A2	94
hsa_circ_0081159	0.006505117	2.5019772	up	chr7	COL1A2	76
hsa_circ_0081160	0.04243376	2.0236626	up	chr7	COL1A2	4
hsa_circ_0081163	0.044002496	2.5647683	up	chr7	COL1A2	10
hsa_circ_0081167	0.0097287	3.1536498	up	chr7	COL1A2	24
hsa_circ_0084720	0.02980772	3.712714	up	chr8	SULF1	4
hsa_circ_0087215	0.023945637	2.500071	up	chr9	ANXA1	
hsa_circ_0089433	0.008899449	2.1055155	up	chr9	COL5A1	364
hsa_circ_0090450	1.11E-04	3.8579054	up	chrX	TIMP1	11
hsa_circ_0090452	0.003296774	2.235864	up	chrX	TIMP1	5
hsa_circ_0091742	0.005013032	3.8982337	up	chrX	BGN	192
hsa_circ_0091743	0.015532353	3.1929755	up	chrX	BGN	181
hsa_circ_0000019	0.020847283	2.5991628	down	chr1	DDI2	0
hsa_circ_0000258	0.042388447	2.687611	down	chr10	PDCD11	0
hsa_circ_0000390	0.01983777	12.444183	down	chr12	FGD4	0
hsa_circ_0000580	0.04103441	4.64374	down	chr14	None	0
hsa_circ_0000615	0.047749862	5.541818	down	chr15	ZNF609	17
hsa_circ_0000642	0.040490992	2.6040976	down	chr15	ZFAND6	2
hsa_circ_0001074	0.030960364	3.0543585	down	chr2	ORC4	0
hsa_circ_0001112	0.018396724	2.6625469	down	chr2	DGKD	0
hsa_circ_0001114	5.34E-04	3.171953	down	chr2	DGKD	0
hsa_circ_0001210	0.03488208	2.5489702	down	chr22	None	1026
hsa_circ_0001216	0.026226409	3.7022111	down	chr22	XBP1	2
hsa_circ_0001438	0.029758973	6.8401494	down	chr4	LARP1B	0
hsa_circ_0001679	0.031237341	8.059054	down	chr7	GLCCI1	2
hsa_circ_0001998	0.025869323	2.7446353	down	chr14	FUT8	0
hsa_circ_0002110	0.018834386	2.806639	down	chr12	AMN1	0
hsa_circ_0002138	0.032208133	3.2452636	down	chr15	USP3	0
hsa_circ_0002190	0.030215895	2.5628076	down	chr7	KLHDC10	1
hsa_circ_0002422	0.037262026	4.9764647	down	chr3	FNDC3B	7
hsa_circ_0002449	0.005389997	4.227186	down	chr5	C5orf32	3
hsa_circ_0002504	0.03395744	2.3386385	down	chr17	ENGASE	4
hsa_circ_0003012	0.0350584	5.214465	down	chr12	SLC41A2	0
hsa_circ_0003201	0.017809883	2.6226783	down	chr4	TBC1D14	0
hsa_circ_0003787	0.009270906	5.055101	down	chr5	RGNEF	2
hsa_circ_0003911	0.0193295	3.0724642	down	chr16	CNOT1	8
hsa_circ_0004689	0.043355685	6.3922377	down	chr1	SWT1	0
hsa_circ_0005028	0.004585072	2.5328124	down	chr3	TSEN2	0
hsa_circ_0005135	0.025206376	2.680219	down	chr19	LOC100506033	1
hsa_circ_0006034	0.01381651	6.038208	down	chr5	SLC12A2	1
hsa_circ_0006511	0.008116972	2.9554853	down	chr2	FARP2	2
hsa_circ_0007315	0.042465463	9.14959	down	chr3	PVRL3	0
hsa_circ_0007538	0.021333938	2.2191985	down	chr1	C1orf27	0
hsa_circ_0007619	0.024274996	6.2581415	down	chr4	LARP1B	0
hsa_circ_0007715	0.02931771	3.3169591	down	chr19	CIRBP	4
hsa_circ_0007754	0.007596167	2.5428722	down	chr13	PCCA	0
hsa_circ_0007840	0.01876142	2.3082905	down	chr15	COX5A	0
hsa_circ_0008962	0.023675451	2.5180075	down	chr5	ELL2	0
hsa_circ_0011092	0.027778216	2.8495584	down	chr1	STX12	0
hsa_circ_0014614	0.046407204	3.509566	down	chr1	DAP3	1
hsa_circ_0015948	0.036354423	2.187048	down	chr1	IPO9	4
hsa_circ_0017445	0.032844365	4.300893	down	chr10	WDR37	0
hsa_circ_0017974	0.003326846	2.0536025	down	chr10	KIAA1217	3
hsa_circ_0020752	0.038835317	3.8155284	down	chr11	None	18
hsa_circ_0020757	0.045137018	3.7709012	down	chr11	None	481
hsa_circ_0020762	0.049438722	3.4821498	down	chr11	None	729
hsa_circ_0020763	0.024364103	7.188659	down	chr11	None	623
hsa_circ_0022351	0.048745744	2.1941059	down	chr11	C11orf9	22
hsa_circ_0023597	0.025558302	2.48522	down	chr11	XRRA1	2
hsa_circ_0025842	0.024139885	6.305559	down	chr12	FGD4	14
hsa_circ_0025847	0.01065637	6.9226236	down	chr12	FGD4	0
hsa_circ_0027969	0.014839446	8.641936	down	chr12	SLC41A2	1
hsa_circ_0027971	0.03575782	7.201395	down	chr12	SLC41A2	1
hsa_circ_0028323	0.03792535	4.157252	down	chr12	TMEM116	0
hsa_circ_0029235	0.04259122	2.0699773	down	chr12	DDX55	0
hsa_circ_0031281	0.016793832	2.7682478	down	chr14	SLC7A8	0
hsa_circ_0031423	0.020782415	2.8116136	down	chr14	SCFD1	2
hsa_circ_0035431	0.00300389	20.631256	down	chr15	CGNL1	46
hsa_circ_0035432	0.011778097	5.5990286	down	chr15	CGNL1	74
hsa_circ_0035435	0.014802514	7.2164187	down	chr15	CGNL1	9
hsa_circ_0035875	0.039918724	2.113102	down	chr15	SPG21	0
hsa_circ_0036510	0.028307231	2.6944883	down	chr15	ZFAND6	1
hsa_circ_0037361	0.027664987	9.436992	down	chr16	C16orf73	0
hsa_circ_0037362	0.027888432	10.314745	down	chr16	C16orf73	0
hsa_circ_0037861	0.017640278	3.1812923	down	chr16	TXNDC11	0
hsa_circ_0037862	0.02676344	5.1893096	down	chr16	TXNDC11	3
hsa_circ_0037863	0.014628965	3.431537	down	chr16	TXNDC11	11
hsa_circ_0038521	0.012550134	3.4246309	down	chr16	CDR2	41
hsa_circ_0039090	0.008517502	2.1161213	down	chr16	SRCAP	467
hsa_circ_0039216	0.032217234	2.741417	down	chr16	GPT2	1
hsa_circ_0039218	0.028291393	3.9539077	down	chr16	GPT2	15
hsa_circ_0039271	0.047586497	2.770235	down	chr16	PHKB	4
hsa_circ_0039658	0.04334005	3.0598512	down	chr16	CNOT1	14
hsa_circ_0039940	0.016690876	2.131468	down	chr16	SLC7A6	0
hsa_circ_0040081	0.013927639	2.3884165	down	chr16	NQO1	0
hsa_circ_0040373	0.021966241	3.0635164	down	chr16	AP1G1	11
hsa_circ_0040388	0.018369772	2.0574872	down	chr16	AP1G1	3
hsa_circ_0041440	0.023598213	2.3573241	down	chr17	RAP1GAP2	53
hsa_circ_0042853	0.049323324	2.8299448	down	chr17	TCONS_00025103	0
hsa_circ_0042968	0.027372789	2.9627814	down	chr17	SUZ12	1
hsa_circ_0045272	0.041974243	2.0751245	down	chr17	ERN1	7
hsa_circ_0047700	0.02186925	5.2570233	down	chr18	ME2	0
hsa_circ_0047785	0.049457386	2.5616474	down	chr18	ATP8B1	6
hsa_circ_0047975	0.044554852	2.6958208	down	chr18	ZNF236	40
hsa_circ_0048201	0.024692174	2.7309535	down	chr19	STK11	110
hsa_circ_0048536	0.049628958	2.0178084	down	chr19	EEF2	63
hsa_circ_0049289	0.034041822	4.4006753	down	chr19	SLC44A2	226
hsa_circ_0051047	0.04167667	2.575785	down	chr19	FCGBP	432
hsa_circ_0051050	0.043033753	3.2892206	down	chr19	FCGBP	3
hsa_circ_0051995	0.020385692	6.4125986	down	chr19	VRK3	14
hsa_circ_0054086	0.032298878	3.289804	down	chr2	HEATR5B	6
hsa_circ_0054186	0.044439603	3.2368143	down	chr2	MAP4K3	0
hsa_circ_0054970	0.030601952	5.295868	down	chr2	SLC1A4	0
hsa_circ_0054971	0.02345602	3.2384007	down	chr2	SLC1A4	0
hsa_circ_0056240	0.008702193	3.180426	down	chr2	PTPN4	0
hsa_circ_0057104	0.047582004	4.0253515	down	chr2	PDK1	1
hsa_circ_0057105	0.03679815	3.6738498	down	chr2	PDK1	2
hsa_circ_0057106	0.047164652	4.9790606	down	chr2	PDK1	1
hsa_circ_0057480	0.030882323	3.0643332	down	chr2	PMS1	1
hsa_circ_0058443	0.008067183	4.725038	down	chr2	ACSL3	1
hsa_circ_0058762	0.012233879	4.721563	down	chr2	DGKD	0
hsa_circ_0058766	0.002712246	3.6002114	down	chr2	DGKD	1
hsa_circ_0058767	0.020062199	2.9678054	down	chr2	DGKD	18
hsa_circ_0058768	0.001202278	2.1620224	down	chr2	DGKD	75
hsa_circ_0058769	0.001321101	2.6733668	down	chr2	DGKD	32
hsa_circ_0058770	0.005304494	5.227343	down	chr2	DGKD	0
hsa_circ_0060108	0.011829588	2.895927	down	chr20	FER1L4	217
hsa_circ_0062721	0.03316098	3.6204636	down	chr22	XBP1	26
hsa_circ_0063555	0.038891237	2.0088708	down	chr22	ACO2	23
hsa_circ_0063561	0.016673693	2.77844	down	chr22	ACO2	35
hsa_circ_0063562	0.03148357	4.083042	down	chr22	ACO2	9
hsa_circ_0063563	0.015282579	3.6569278	down	chr22	ACO2	9
hsa_circ_0063567	0.019898534	2.1735334	down	chr22	ACO2	0
hsa_circ_0065143	0.040677425	2.0057971	down	chr3	SETD2	57
hsa_circ_0066873	0.048354574	3.8894832	down	chr3	TIMMDC1	0
hsa_circ_0066877	0.034795098	3.1437237	down	chr3	TIMMDC1	0
hsa_circ_0066971	0.011570533	5.8554688	down	chr3	EAF2	0
hsa_circ_0067450	0.01625185	7.951924	down	chr3	PPP2R3A	6
hsa_circ_0068032	0.035557568	4.645977	down	chr3	NAALADL2	5
hsa_circ_0069113	0.027285358	2.006047	down	chr4	TBC1D14	0
hsa_circ_0069114	0.002569319	2.1330538	down	chr4	TBC1D14	0
hsa_circ_0070936	0.04316312	2.6107051	down	chr4	LARP1B	7
hsa_circ_0071107	0.006979433	3.2499113	down	chr4	ARHGAP10	2
hsa_circ_0071321	9.78E-04	4.8015165	down	chr4	FGA	3
hsa_circ_0072309	0.002072746	7.3090854	down	chr5	LIFR	0
hsa_circ_0072789	0.009060388	3.3077662	down	chr5	MARVELD2	9
hsa_circ_0072997	0.048613824	2.7817066	down	chr5	RGNEF	1
hsa_circ_0072998	0.015955605	5.7427354	down	chr5	RGNEF	0
hsa_circ_0073006	0.014696714	2.9636796	down	chr5	RGNEF	35
hsa_circ_0073035	0.013912499	2.19241	down	chr5	HMGCR	0
hsa_circ_0073244	0.004625377	4.716427	down	chr5	EDIL3	0
hsa_circ_0073582	0.019003673	3.4118779	down	chr5	EPB41L4A	0
hsa_circ_0073763	0.029358098	2.9471374	down	chr5	SLC12A2	2
hsa_circ_0073768	0.029927656	4.6921773	down	chr5	SLC12A2	1
hsa_circ_0073769	0.005703302	3.6955311	down	chr5	SLC12A2	1
hsa_circ_0073770	0.008143293	6.911025	down	chr5	SLC12A2	1
hsa_circ_0073771	0.031154156	4.1421623	down	chr5	SLC12A2	1
hsa_circ_0073772	0.031598363	3.3706727	down	chr5	SLC12A2	1
hsa_circ_0073955	0.03778204	4.6350393	down	chr5	SEC. 24 A	21
hsa_circ_0074239	0.002184121	6.5593286	down	chr5	C5orf32	13
hsa_circ_0075447	0.043269653	4.7036705	down	chr6	GMDS	0
hsa_circ_0075538	0.018290881	4.5513425	down	chr6	F13A1	19
hsa_circ_0076303	0.0419991	2.85898	down	chr6	PGC	16
hsa_circ_0076304	0.014676455	31.56073	down	chr6	PGC	35
hsa_circ_0076305	0.029925665	125.95259	down	chr6	PGC	49
hsa_circ_0076307	0.025423396	9.498694	down	chr6	PGC	1
hsa_circ_0077168	0.04191364	2.536258	down	chr6	BCKDHB	1
hsa_circ_0077736	0.048207846	6.2890162	down	chr6	CEP85L	2
hsa_circ_0082915	0.022677844	2.0698295	down	chr7	SLC4A2	2
hsa_circ_0083027	0.02473872	3.3468018	down	chr7	MLL3	8
hsa_circ_0084925	0.017582932	5.8715267	down	chr8	KIAA1429	0
hsa_circ_0088633	0.002749995	2.1568172	down	chr9	GARNL3	0

### CircRNA-miRNA network

**Figure 4:**
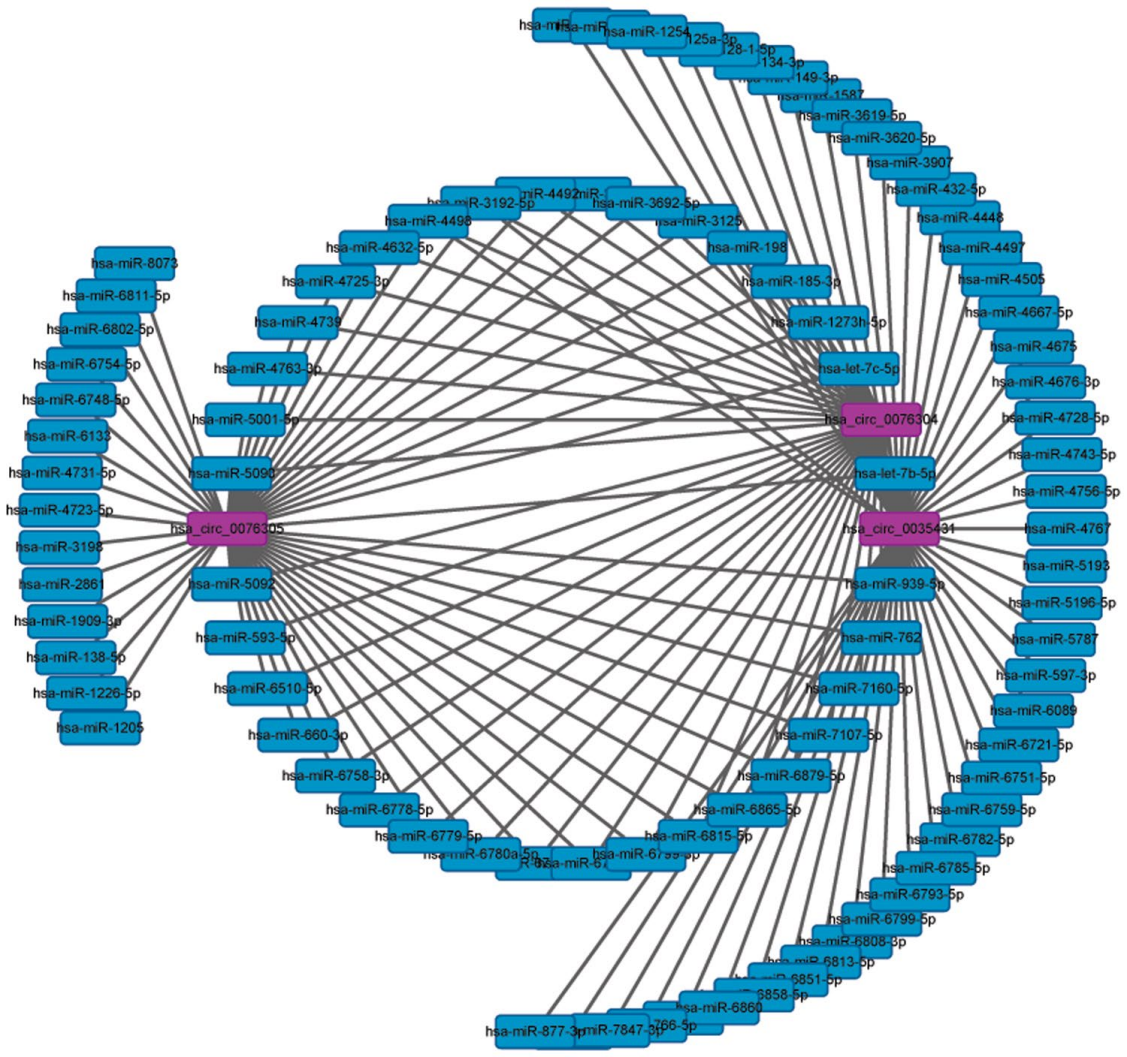
Represent circRNA-miRNA network. This network was based on the expression profile results and the related software. The 3 dysregulated circRNAs, hsa_circ_0076304, hsa_circ_0035431, and hsa_circ_0076305 (purple red nodes) having the highest magnitude of change, were predicted to be functionally connected with their targeted miRNAs in the network.

The 3 circRNAs with most robust differential expression were used to construct a  represent circRNA-miRNA network. The CBC1 and CBC2 probes identified a total of 207 differentially expressed circRNAs; among these circRNAs, hsa_circ_0076304, hsa_circ_0035431, and hsa_circ_0076305 had the highest magnitude of difference. Figure 4 illustrates the interaction of the 3 circRNAs with miRNA.

## Discussion

CircRNAs are recently identified as disease-related and ubiquitously expressed noncoding RNAs, that can act as sponges of miRNAs and affect the expression of parent gene^11–14^. During the past several years, increasing evidence suggested that circRNAs play a vital role in cancer development and may be used as novel biomarkers^15–18^. By comparing circRNAs expression profiles in parental cell line and established cell line with radioresistant effects, Su *et al*. found that dysregulated circRNAs are related to the progression of radiation resistance in esophageal cancer cells^19^. Huang *et al*.^20^ reported that dysregulated lncRNAs and circRNAs are linked to the development of bladder cancer. They identified that several hundreds of circRNAs showed altered expression in bladder cancer tissues as analyzed by the expression profiles of 4 paired cancer and para-carcinoma tissues. They postulated that several of the dysregulated circRNAs are functional molecules and contribute to bladder cancer tumorigenesis. In the present study, 207 circRNAs were found to be differentially expressed  between GC and non-cancerous tissues by both CBC1 and CBC2 probes in the microarray chip. Hsa_circ_0044516 had the highest magnitude of upregulation, whereas hsa_circ_0076305 had the highest magnitude of downregulation. The randomly selected 7 circRNAs that were significantly altered were further verified by qRT-PCR. These results conformed the validity of the microarray findings.

Some of the previously identified circRNAs are implicated  to be associated with tumorigenesis and malignant behavior of cancer cells, such as uncontrolled growth, proliferation, migration, and invasion. For example, Hsa_circ_0067934 has been shown to be upregulated in esophageal squamous cell carcinoma (ESCC)^21^, and associated with poor tumor differentiation. In their findings, hsa_circ_0067934 was able to increase ESCC cell proliferation, migration, and cell cycle progression^21^. Xu *et al*.^22^ showed that patients with hepatocellular carcinoma (HCC) with higher expression level of circular RNA ciRS-7 (Cdr1as) in cancerous tissues had shorter median recurrent time than those with lower circRNA expression. Additionally, Cdr1as was related to the high hepatic microvascular invasion (MVI) in HCC, and the mechanism may be associated with its potential activity as the sponge of miR-7. Therefore, the study concluded that Cdr1as might be a novel biomarker and treatment target for MVI.

CircRNAs can regulate the transcription of parent genes. In the present study, we identified 116 corresponding linear mRNAs. GO and pathway enrichment analysis showed that these mRNAs are involved in critical pathways associated with cancer, including the PI3K-AKT pathway. Previously studies have shown that activation of the PI3K-AKT pathway promote cancer cell growth and proliferation^23, 24^. One of the potential targets of hsa_circ_0039090, hsa-let-7c-5p is  associated with stage I endometrioid endometrial carcinoma progression potentially through regulation of cell cycle pathway^25^. Hsa-miR-107, one of the targets of several dysregulated circRNAs identified in the present study, is widely confirmed to be associated with cancers^26–30^, which is the downstream target of circTCF25, and the interaction between this circRNA with miR-107 and miR-103a-3p leads to increased proliferation and migration of bladder cancer cells^31^.

CircRNA-miRNA network is a widely accepted approach for exploring the function of dysregulated circRNAs and the interaction between these two types of non-coding RNAs. Hence, this network was constructed based on the microarray data. Among altered circRNAs, hsa_circ_0076304, hsa_circ_0035431, and hsa_circ_0076305 had the highest magnitude of difference. Concurrently, the potential links between them and the most important targeted miRNAs were established.

In summary, this study provided a preliminary landscape of circRNA differential expression in GC vs. non-GC. Further studies are required to explore their potential as biomarkers for GC as well as their pathologic role.

## Electronic supplementary material

Table S1

Table S2

Table S3

Table S4
