# Supplementary material for: Circular RNAs expression profiles in human gastric cancer
Source: Sci Rep. 2017 Aug 22;7:9060. doi: 10.1038/s41598-017-09076-6 (PMC5567231; doi:10.1038/s41598-017-09076-6)
Supplement: Supplementary file 1 — Table S1 [file 41598_2017_9076_MOESM1_ESM.pdf]

Circular RNAs expression profiles in human gastric cancer

Yuan Dang, Xiaojuan Ouyang, Fan Zhang, Kai Wang, Youdong Lin, Baochang Sun, Yu Wang, Lie Wang, Qiaojia Huang

Table S1. Primers used for qRT-PCR verification of the 7 random selected circRNAs

circRNA Name	Forward primer (5'->3')	Reverse primer(5'->3')
hsa_circ_0081146	TGGCAGCCAGGGGGAAC	TCCAGAAGGACCTCGGCTTC
hsa_circ_0084720	AGGACTCAGCCCCACAGTTT	TGTCTCCGTCTCCTGATTTCTG
hsa_circ_0054971	GGCTCCGAAGGAGAAGACCT	ACGGAAAGCTGCAACCACAA
hsa_circ_0057104	TGGATTGCCCATATCACGTCT	GGCTGTCCTGGTGATTTTGCT
hsa_circ_0058766	AGCATCGACTCCGATGTGTG	GAGGCCAGTGTGGTCCACTT
hsa_circ_0060108	GATGCCCAAGGGACCTCAAG	AAAGGGGAGGGTCTGCGAAT
hsa_circ_0063561	GAGACCGACTACCTGACGGG	GGCCGGTTCAGTCCCTTTG
GAPDH	AGCCACATCGCTCAGACAC	GCCCAATACGACCAAATCC
